# Linking Downbeat Nystagmus and Aquaporin-4-Positive Neuromyelitis Optica: A Closer Look at Their Hidden Relationship

**DOI:** 10.7759/cureus.71927

**Published:** 2024-10-20

**Authors:** Isabella Canut, Blaire Cote, Charles Maitland

**Affiliations:** 1 Medicine, Philadelphia College of Osteopathic Medicine, Suwanee, USA; 2 Pediatrics, University of Florida Health, Gainesville, USA; 3 Neuro-ophthalmology, Department of Clinical Sciences, Florida State University College of Medicine, Tallahassee, USA

**Keywords:** aquaporin-4 (aqp4) antibody, autoimmune demyelination, demyelination, downbeat nystagmus, immunotherapy, long-segment myelitis, neuromyelitis optica, oculomotor dysfunction, optic neuropathy, uplizna (inebilizumab)

## Abstract

Neuromyelitis optica (NMO), an autoimmune disease, typically presents with loss of vision and myelopathic signs. NMO may be associated with antibodies selective for aquaporin-4 (AQP4), a water channel located within the optic nerves and spinal cord.AQP4 is distributed in the periventricular region, corpus callosum, magnocellular nuclei of the hypothalamus, and brain stem.Downbeat nystagmus (DBN) is associated with structural, metabolic, and autoimmune disorders, but is rarely reported in NMO.We treated a 33-year-old female with known AQP4-positive NMO who presented with new-onset DBN. We review the literature and discuss possible mechanisms and lesion locations hypothetically responsible for the symptoms and signs.

## Introduction

Neuromyelitis optica (NMO) is classically associated with aquaporin-4 (AQP4) antibody (AQP4-IgG), a water channel protein found expressed in the end-feet of astrocytes in the central nervous system (CNS) that facilitates two-way water movement across cellular membranes. AQP4 is distributed in specific areas, including the periventricular region, corpus callosum, magnocellular nuclei of the hypothalamus, medulla oblongata, periaqueductal gray, and cerebellum [1]. Downbeat nystagmus (DBN) is an involuntary, repetitive eye movement in which there is a slow upward drift of the eyes followed by a fast downward corrective movement. DBN is a key clinical sign of cerebellar or brainstem dysfunction. Its causes are diverse, ranging from structural abnormalities (damage to the flocculus or nodulus of the cerebellum, medullary brain lesions, foramen magnum lesions, Arnold-Chiari malformations type I or II) to metabolic, toxic, drug-induced, trauma, and autoimmune factors. However, DBN is seldom seen in NMO [2]. Animal studies suggest that AQP4-IgG autoantibodies originate in the peripheral nervous system and subsequently bind to astrocytic foot processes in the CNS. This attachment leads to infiltration, cell damage mediated by complement, and the demise of astrocytes. Subsequently, astrocyte death triggers demyelination and neuronal cell death [3].

NMO is relatively rare, with an estimated prevalence of approximately one to two per 100,000 people worldwide [4]. It predominantly affects females, with a female-to-male ratio of about 9:1 [4,5]. While it can occur at any age, the peak onset is typically between the ages of 35 and 45 years. Additionally, NMO is more common in certain ethnic groups, such as individuals of African or Asian descent [4].

Typically, NMO attacks manifest as optic neuritis, with or without long-segment transverse myelitis, and neurological deficits tend to emerge gradually over a span of days. Demyelination of neurons results in varied clinical manifestations depending on the specific neuronal pathways involved. In cases of NMO, visual, motor, and sensory neurons may be affected. Within five years of disease onset, approximately 50% of patients develop significant visual and/or motor deficits, leading to substantial functional impairment [6]. Additional symptoms include intractable hiccups, persistent nausea and vomiting, and acute encephalopathy, particularly in patients with brainstem involvement.

Evaluation of patients with NMO typically includes cerebrospinal fluid (CSF) studies, ophthalmologic and neurologic examinations, and neuroimaging. Magnetic resonance imaging (MRI) allows clinicians to examine normal and pathological anatomy within the brain. However, in patients with NMO, brain and brainstem lesions are not always detected through MRI imaging [7]. To diagnose NMO, other conditions, particularly multiple sclerosis (MS), anti-myelin oligodendrocyte glycoprotein (MOG) antibody-associated disease, and sarcoidosis, need to be excluded. NMO was historically misclassified as a subtype of MS due to overlapping clinical features. However, NMO primarily affects the spinal cord and optic nerves, with occasional brainstem involvement, while MS predominantly involves the brain, spinal cord, and optic nerve. A clinical presentation of one or more severe episodes of optic neuritis (ON) in conjunction with transverse myelitis raises suspicion for NMO [8]. Cognitive dysfunction, such as impairments in memory, reasoning, and problem-solving, is frequently observed in MS but is generally absent in NMO. Furthermore, the two conditions exhibit distinct MRI characteristics. Brain MRI in NMO patients typically shows minimal or no lesions, whereas spinal MRI often reveals longitudinally extensive lesions spanning three or more vertebral segments [9]. In contrast, MS lesions are usually smaller and more widespread throughout the CNS. CSF analysis in NMO often reveals pleocytosis, with leukocyte counts exceeding 50 cells/µL, sometimes accompanied by neutrophils. Serological testing for AQP4 antibodies is positive in approximately 70% of NMO patients. AQP4 is a protein in astrocytes, a type of cell that helps the nerves in the CNS function properly. Differentiating NMO from MS is crucial, as standard MS treatments, such as beta interferons, can exacerbate NMO and lead to increased relapse rates [8,9].

Preferred treatments for acute attacks of NMO include IV corticosteroids to reduce inflammation, intravenous immunoglobulin (IgG), and plasmapheresis to lower the level of anti-AQP4 antibodies in the blood. Long-term management often involves ongoing immunosuppressive agents such as eculizumab, inebilizumab-cdon, ravulizumab-cwvz, and satralizumab-mwge, which are FDA-approved for anti-AQP4-positive NMO [10].

As previously mentioned, DBN is typically associated with various pathologies, including skull base lesions, cerebellar degeneration syndromes, medication toxicities, FGF14 gene variations, and metabolic disorders, including GAD65 syndrome, or without an identified cause. Investigating localization patterns in 91 patients diagnosed with DBN, Yee observed cerebellar involvement in 88% of cases through clinical evaluations. Yee further delineated that lesions affecting the cerebellar regions responsible for horizontal tracking and visual-vestibulo-ocular interactions were predominantly implicated. Conversely, animal studies have suggested a potential medullary origin for DBN [11].

This article was previously presented as a meeting abstract at the 2024 AAN Annual Meeting on April 16, 2024.

## Case presentation

A 33-year-old female initially presented with blurry vision. This visual disturbance was the first symptom that prompted her to seek medical attention. She was diagnosed with relapsing-remitting multiple sclerosis and treated with IV methylprednisolone with partial recovery, followed by an infusion of Tysabri three weeks later. A month later, she developed pronounced pain and numbness in her lower extremities, significantly impacting her mobility and overall quality of life. She was found to have positive AQP4 antibody NMO. As the disease progressed, she exhibited a staggered onset of optic neuropathy and long-segment myelitis, both characteristic of AQP4 NMO.

Sequential examinations of her visual system indicated significant atrophy in both eyes (20/400 in the right eye (OD) and 20/600 in the left eye (OS)), signifying severe and irreversible optic nerve damage. Additionally, she exhibited saccadic pursuit, indicating rapid, jerky eye movements while tracking moving objects. Notably, these movements were devoid of nystagmus or overshoot dysmetria.

The patient underwent various immunologic therapies in an attempt to manage her condition and alleviate symptoms. Treatments included Cytoxan (cyclophosphamide), Rituxan (rituximab), Copaxone (glatiramer acetate), and Tysabri (natalizumab), all of which are known to be efficacious in preventing relapses with NMO spectrum disorder. Despite these interventions, her condition progressed, leading her to participate in an investigational trial involving Uplizna (inebilizumab-cdon). Uplizna targets CD19-expressing B cells, which are a pathogenic driver of NMO, leading to the depletion of B cells. The most common side effects are arthralgia and urinary tract infections.

Two years into the trial, specific clinical signs were observed, including bidirectional horizontal nystagmus, right hypertropia, and oblique DBN more pronounced in the left downgaze than in the right downgaze. Ocular movements (ductions and versions) remained fully intact, with no evidence of oculo-lateral pulsion. The head thrust test yielded negative results, indicating normal vestibulo-ocular reflex (VOR) function.

**Figure 1:**
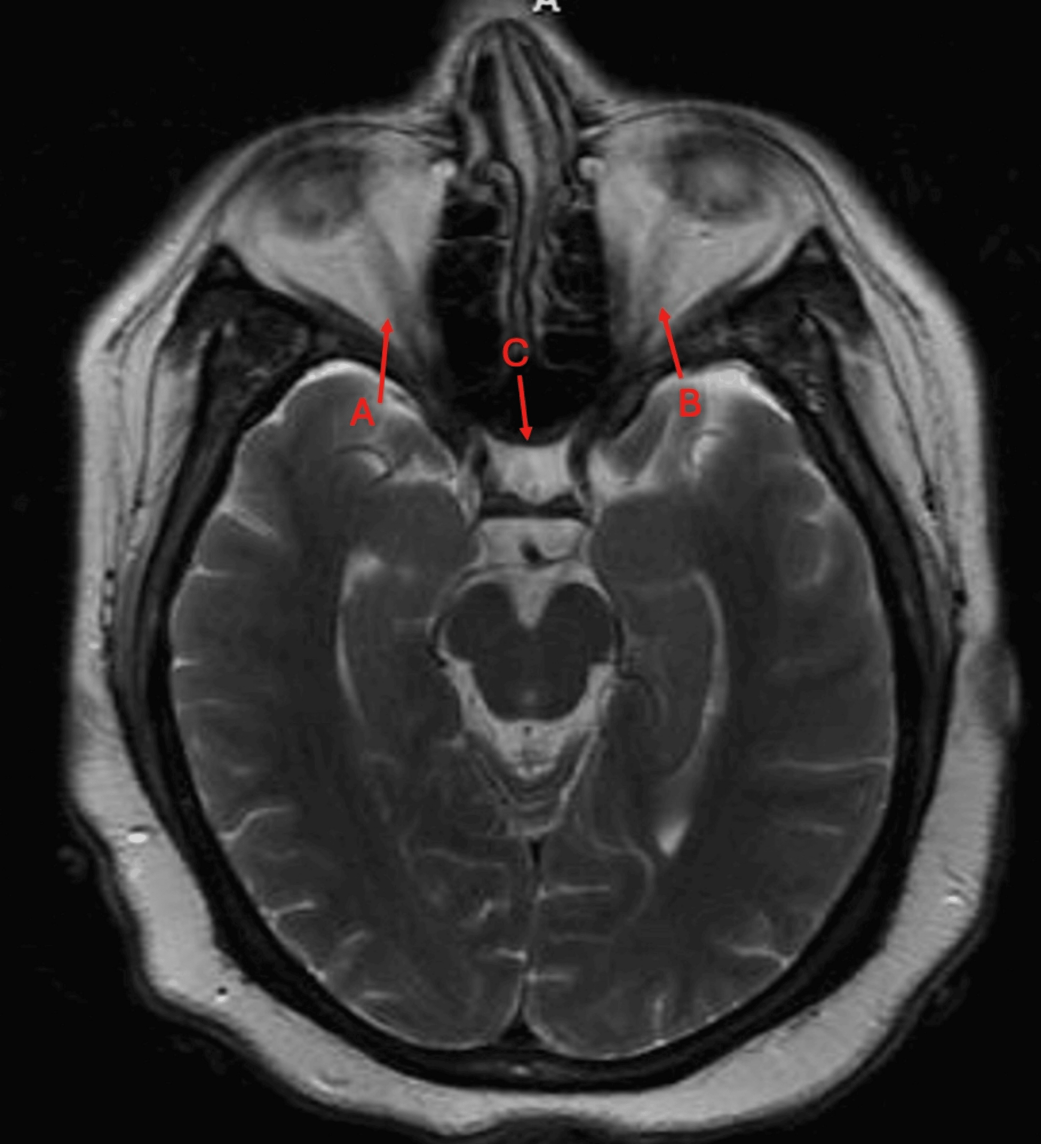
Axial T2-weighted MRI of the brain in a patient with neuromyelitis optica. Arrows A and B indicate the optic nerve. The opaque (white) regions within the nerve are signs of inflammation, i.e., optic neuritis. Arrow C is pointing to the optic chiasm. MRI shows hyperintense signal abnormalities in the optic nerves, most pronounced bilaterally, consistent with inflammation or prior optic neuritis. The findings suggest optic nerve involvement, a common characteristic of neuromyelitis optica. No acute demyelinating lesions are visible within the brainstem or cerebellum at this level. The ventricles appear symmetrical and there are no signs of significant mass effect or midline shift.

MRI of the brain and orbits revealed non-enhancing atrophy of the optic nerves and chiasm, consistent with chronic damage (Figure 1). No other abnormalities were detected on the MRI.

**Figure 2:**
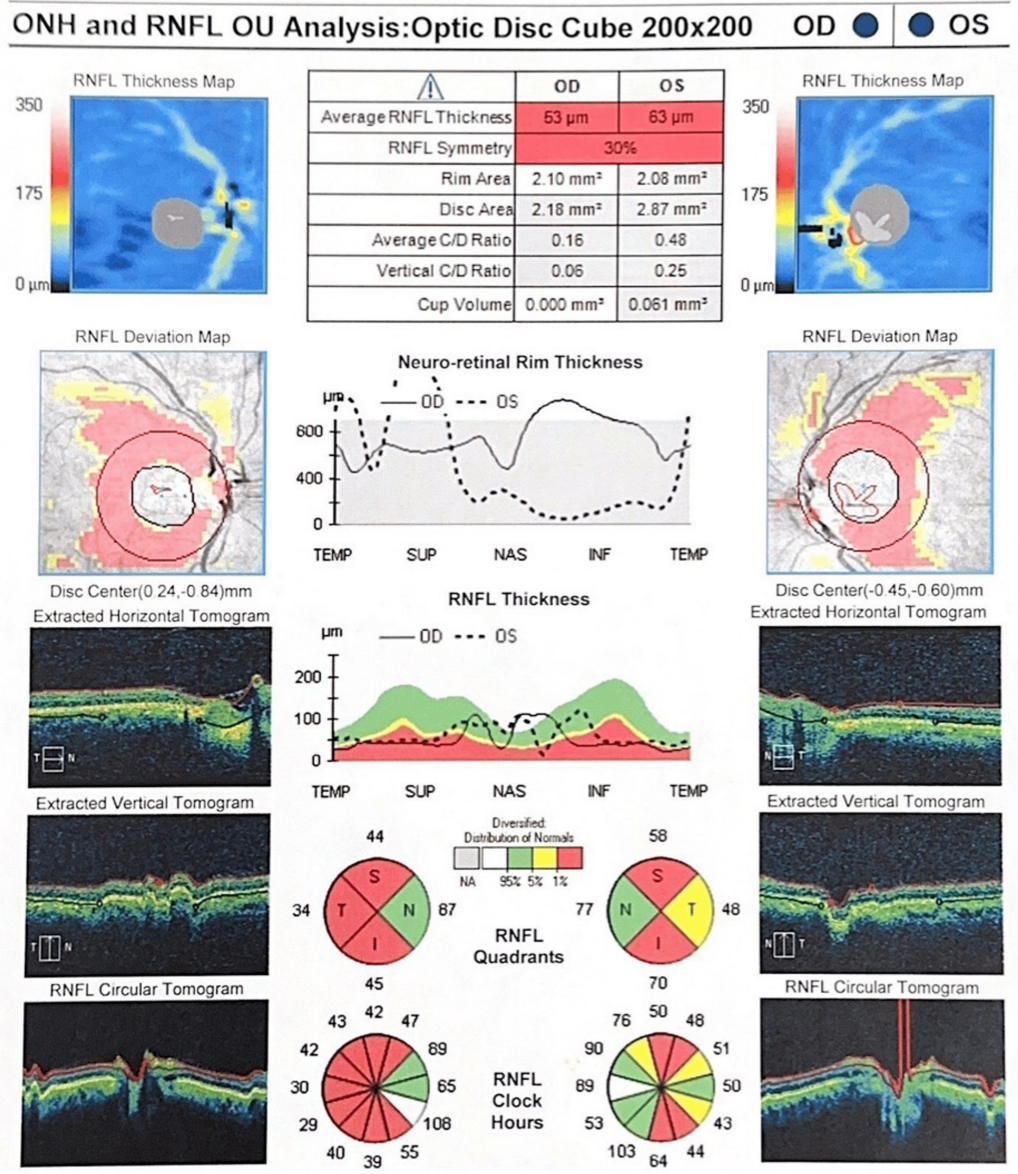
Optical coherence tomography (OCT) of the optic nerve head (ONH) and retinal nerve fiber layer (RNFL) in a patient with neuromyelitis optica (NMO). The OCT analysis of the ONH and RNFL demonstrates significant thinning, particularly in the right eye (OD), consistent with optic neuritis secondary to NMO. The average RNFL thickness in the right eye is markedly reduced to 53 µm, most prominent in the superior and inferior quadrants, indicating substantial axonal loss. The left eye (OS) shows less severe thinning, with an average RNFL thickness of 63 µm. OU: oculus uterque (both eyes).

Optical coherence tomography (OCT) findings (Figure 2) demonstrated substantial thinning of the retinal nerve fiber layer (RNFL) in the OD, with an average thickness of 53 µm, predominantly affecting the superior and inferior quadrants. The OS showed comparatively less thinning, with an average RNFL thickness of 63 µm. This asymmetry between the eyes highlights the cumulative impact of recurrent optic neuritis episodes in NMO, which often lead to significant axonal loss and long-term visual impairment.

These findings are consistent with the patient’s clinical history and emphasize the destructive nature of NMO on the optic nerves, further supporting the diagnosis of NMO-related optic neuropathy.

This case highlights the complexity and severity of AQP4 NMO and underscores the challenges in managing this debilitating disease despite extensive treatment efforts.

## Discussion

Pathophysiology

In NMO, the binding of AQP4-IgG to astrocytes disrupts their normal function, impairing their ability to regulate extracellular potassium levels and maintain blood-brain barrier integrity. This disruption leads to edema and inflammation, particularly around blood vessels. The inflammatory milieu contributes to astrocyte injury and death, triggering demyelination and neuronal cell loss. The subsequent loss of myelin sheaths and axonal damage further perpetuates the neuroinflammatory process, resulting in irreversible neurological deficits [12-14]. Pro-inflammatory cytokines and chemokines released during this process sustain the autoimmune response and attract additional immune cells to the site of injury, exacerbating tissue damage [15].

The involvement of the brainstem and cerebellum is particularly pertinent to the development of DBN in NMO [16]. The brainstem houses the vestibular nuclei, which are crucial for processing and integrating vestibular and proprioceptive information necessary for gaze stabilization. Inflammation and demyelination within the brainstem, especially affecting the vestibular nuclei and their associated tracts, disrupt the normal function of these structures. This disruption impairs the VOR pathways, leading to the ocular motor dysfunction characteristic of DBN [16,17].

Additionally, the cerebellum, through its connections with the brainstem and vestibular system, plays a critical role in the coordination and fine-tuning of eye movements. Inflammation and demyelination affecting the cerebellar pathways, particularly the vestibulocerebellar tracts and the flocculonodular lobe, significantly impact the cerebellum’s ability to modulate eye movement and maintain visual stability. Damage to these cerebellar structures contributes to DBN by impairing the integration of vestibular and visual inputs.

DBN arises from the disruption of neural circuits involved in vertical gaze control. Impairment of the superior colliculus, which is involved in the generation of vertical saccades, along with dysfunction of the medial vestibular nuclei and cerebellar flocculus, results in an imbalance in vertical gaze control mechanisms. This imbalance manifests as DBN.

In summary, the pathophysiology of NMO involves a complex interplay of autoantibodies, complement activation, inflammation, and immune cell infiltration, leading to astrocyte injury, demyelination, and neuronal damage. This damage, particularly in the brainstem and cerebellar regions, contributes to the development of DBN in affected individuals.

Up to 95% of cases of NMO are relapsing, meaning they have more than one attack. In contrast, monophasic NMO occurs in 5-10% of cases and involves a single attack. To be diagnosed with monophasic NMO, the patient must be attack-free for five or more years. Patients with monophasic NMO typically appear to be AQP4-IgG-seronegative compared to those with relapsing NMO [18].

Cases in the literature

Hage et al. [2] reported four cases of DBN in patients with AQP4-antibody-positive NMO. MRI data were available for three of the cases, revealing lesions in the dorsal medulla, caudal medullary region, and periaqueductal gray area. Notably, a 37-year-old female with a 10-year history of NMO developed DBN two months after discontinuing weekly plasma exchange therapy due to infectious spondylitis. The authors speculated that DBN may result from dysfunction in the cerebellovestibular inhibitory pathways between the flocculus and the superior vestibular nucleus, leading to the disinhibition of elevator motoneurons in the superior vestibular nucleus.

Shirai et al. [19] described a case of a 48-year-old man in Japan who presented with nausea, hiccups, orthostatic hypotension, DBN, rightward gaze palsy, and right partial hemifacial palsy. Initial brain MRI showed a dorsal medullary lesion along the fourth ventricle and a subarachnoid hemorrhage. He was found to be AQP4 positive, diagnosed with NMO, and started on steroid therapy. Follow-up MRI showed medullary lesion improvement. The MRI abnormalities in the dorsal medullary region of this patient suggested disruption of the blood-brain barrier and delicate blood vessels due to elevated levels of AQP4. This disruption could potentially lead to subarachnoid hemorrhage and brain edema, given AQP4's role in astrocyte-mediated regulation of brain water balance.

Yee [11] examined ocular motor function in 90 patients with NMO spectrum disorder, and gaze-evoked nystagmus was observed in 23 patients (25.6%), with upbeat or downbeat nystagmus identified in eight patients (9.3%). Eye movement abnormalities were noted in 38% of the patients during clinical examination, and abnormalities in the quantitative saccadic eye test were found in 67% of the patients. Although no additional cases of DBN associated with AQP4 have been reported in the literature, the relationship between DBN and NMO warrants further investigation.

Similarities

Presence of Downbeat Nystagmus

Along with previously reported cases, our patient developed DBN. Hage et al. [2] observed DBN in a 37-year-old patient following plasma exchange discontinuation, suggesting a potential trigger of DBN in the context of fluctuating immune modulation.

Severe Optic Neuropathy

Optic nerve involvement is a hallmark of AQP4 NMO. Similar to our patient, optic atrophy and long-segment myelitis were common across case reports, showing the aggressive nature of the disease in visual pathways.

Cerebellovestibular Dysfunction Hypothesis

The hypothesis that DBN in AQP4 NMO results from cerebellovestibular disinhibition aligns with the ocular motor findings in our case. Although our patient lacked MRI evidence of brainstem lesions, the presence of DBN supports a similar underlying mechanism involving the vestibular and cerebellar circuits.

Differences

Timing and Triggers of DBN

In our case, DBN emerged during a trial of Uplizna, while in the cases reported by Hage et al. [2], DBN occurred after discontinuation of plasma exchange. This temporal difference suggests that immune modulation may variably influence the onset of DBN in AQP4 NMO.

Additional Ocular Findings

Our patient exhibited bidirectional horizontal nystagmus and right hypertropia, which were not described in the other reports [2,6,18]. Furthermore, our patient's VOR testing was negative, indicating normal vestibulo-ocular function, while other cases noted brainstem lesions, suggesting more widespread neurological involvement.

Absence of Brainstem Lesions

Unlike the cases of Hage et al. and Shirai et al., [2,19], which showed lesions in the dorsal medulla, our patient’s MRI was devoid of brainstem or cerebellar abnormalities, highlighting the variability in neuroimaging findings in AQP4 NMO with DBN.

Systemic Involvement

Shirai et al. [19] reported systemic features such as nausea, orthostatic hypotension, and hemifacial palsy, which were absent in our case. This discrepancy points to the heterogeneous clinical spectrum of AQP4 NMO.

These studies have several limitations. Firstly, many studies in this field have small sample sizes, limiting the generalizability of their findings. In this particular case, it is not known whether the damage associated with the nystagmus was caused by the NMO or the experimental drug trial. While these studies contribute valuable insights, it is essential to acknowledge their limitations and consider how they may impact the interpretation of the findings. Addressing these limitations in future research is crucial for advancing our understanding of the pathophysiology of NMO and its associated symptoms.

## Conclusions

The occurrence of DBN in this AQP4-positive patient, despite the absence of MRI-confirmed lesions, suggests alternative mechanisms of DBN in NMO, potentially involving undetectable functional or structural changes. This case highlights the importance of considering NMO in patients with atypical oculomotor symptoms, even when radiological findings are negative. Further research, including longitudinal and animal studies, is needed to elucidate these mechanisms, track disease progression, and optimize the diagnosis and management of NMO.
